# Transcriptome combined with Mendelian randomization to screen key genes associated with mitochondrial and programmed cell death causally associated with diabetic retinopathy

**DOI:** 10.3389/fendo.2024.1422787

**Published:** 2024-11-20

**Authors:** Shule Jiang, Xuemei Han

**Affiliations:** Department of Ophthalmology, The First Affiliated Hospital of Zhejiang Chinese Medical University (Zhejiang Provincial Hospital of Chinese Medicine), Hangzhou, Zhejiang, China

**Keywords:** diabetic retinopathy, immune infiltration, Mendelian randomization, programmed cell death, mitochondria

## Abstract

**Background:**

Mitochondrial dysfunction in the retina can induce apoptosis of retinal capillary cells, leading to diabetic retinopathy (DR). This study aimed to explore key genes related to programmed cell death (PCD) and mitochondria in DR via bioinformatic analysis.

**Methods:**

A differential analysis was performed to identify differentially expressed genes (DEGs) between DR and control samples using the GSE94019 dataset from the Gene Expression Omnibus (GEO) database. Pearson correlation analysis was then utilized to select genes linked to mitochondrial function and PCD (M-PCD). Candidate genes were identified by overlapping DR-DEGs and M-PCD genes, followed by functional annotation. Mendelian randomization (MR) analysis was employed to identify genes with causal relationships to DR. Key genes were identified through protein-protein interaction (PPI) analysis using six algorithms (DEgree, DMNC, EPC, MCC, Genes are BottleNeck, and MNC) within Cytoscape software. The expression patterns of these genes were validated using GSE94019 and GSE60436 datasets, as well as RT-qPCR. Enrichment analysis provided insights into the function and pathways of these key genes in DR. Differential immune cell profiles were determined via immune infiltration analysis, followed by exploring the relationships between immune cells, cytokines, and the identified genes. Correlations between key genes and apoptosis genes were also examined. *In vivo* experiments using RT-PCR, immunohistochemistry (IHC), and western blot analysis confirmed that MYC and SLC7A11 expression was significantly elevated in DR rat retinal tissues.

**Results:**

From 658 candidate genes, 12 showed significant causal associations with DR. MYC and SLC7A11 were particularly notable, showing upregulated expression in DR samples and involvement in apoptosis and diabetes-related pathways. These genes were significantly associated with apoptotic genes and correlated positively with altered immune cell types and cytokines, suggesting a link between immune response and DR pathogenesis. *In vivo* findings confirmed that MYC and SLC7A11 expression was elevated in DR rat retinal tissues.

**Conclusion:**

Key genes (MYC and SLC7A11) associated with mitochondrial function and PCD in DR were identified, offering insights into DR’s pathological mechanisms and potential targets for diagnostic and therapeutic strategies.

## 1 Introduction

Diabetic retinopathy (DR) is a leading cause of vision impairment and blindness worldwide, affecting both working-age adults and the elderly (1, 2). It is estimated that by 2030, the number of individuals affected by DR will rise to 191 million (2). DR represents a major manifestation of diabetic microvascular complications, characterized by alterations in retinal endothelial vascular structure and breakdown of the blood-retinal barrier (3). In its early stages, DR manifests through endothelial cell and pericyte apoptosis, vascular leakage, and leukostasis, potentially progressing to microaneurysms, retinal vein occlusion, diabetic macular edema, and proliferative DR, all of which pose serious threats to vision (4).

Research indicates that various interconnected biochemical pathways drive DR symptom development, especially under hyperglycemic conditions. Mitochondrial dysfunction in the retina increases superoxide levels, accelerating cytochrome C release, capillary cell apoptosis, and DNA damage (5) The NOD-like receptor family pyrin domain-containing 3 (NLRP3) inflammasome has been identified as a pathogenic factor in retinal cells (6). In the diabetic environment, the NLRP3 inflammasome is activated through pathways such as reactive oxygen species (ROS) and ATP, leading to the secretion of pro-inflammatory cytokines interleukin-1β (IL-1β) and interleukin-18 (IL-18), subsequently inducing apoptosis (7).

A core theory in DR pathogenesis is that enhanced oxidative stress under diabetic conditions damages the retinal microvasculature, triggering microvascular complications (8). Mitochondrial-derived oxidative stress disrupts the homeostasis of various retinal cell types, exacerbating retinal damage. This cascade of events illustrates how diabetes, through oxidative stress, progressively destabilizes retinal homeostasis, ultimately leading to DR development and progression (9).

Despite extensive clinical research on DR, its pathogenic mechanisms are not fully elucidated. Hence, exploring the pathogenesis of DR and its molecular key genes for diagnosis and treatment is crucial for improving patient outcomes.

Mitochondria, key organelles coordinating the biosynthesis of lipids, amino acids, and nucleotides and essential for energy metabolism processes such as the tricarboxylic acid cycle, electron transport system, and β-oxidation of fatty acids, play a significant role in DR (10). Mitochondrial biogenesis is the process of increasing mitochondrial quantity, which relies on the coordinated action of cellular signaling molecules, molecular chaperones, and transcription factors, and is regulated upstream by oxidative stress (5). Mitochondrial dysfunction in DR is evidenced by disrupted mitochondrial dynamics, mitochondrial DNA damage, and increased oxidative stress, leading to aberrant cellular energy metabolism and activation of apoptotic pathways (11). Moreover, these mitochondrial alterations not only affect the survival of retinal cells but may also promote inflammatory responses and neovascularization, exacerbating the progression of DR.

Programmed cell death (PCD), a process where cells actively proceed toward death through a series of ordered molecular events, includes types such as apoptosis, necrosis, and autophagy (12, 13). The intrinsic pathway of PCD is initiated by increased permeability of the mitochondrial outer membrane and activation of apoptotic signaling, while oxidative stress is an early event in the pathogenesis of DR, with PCD typically occurring as a later and secondary event (14). In the onset of DR, apoptosis, in particular, has a pivotal role in damaging retinal neurons and vascular cells. Excessive activation of apoptosis leads to the over-death of retinal cells, thereby disrupting retinal structure and function (15, 16). Additionally, PCD is associated with inflammatory responses and vascular abnormalities in DR, contributing to the exacerbation of the condition (16). Although the significance of mitochondrial dysfunction and PCD in DR is well recognized, the precise mechanisms by which they regulate the pathogenesis of DR and their interplay remain to be clarified. Therefore, a deeper exploration of these molecular mechanisms will not only uncover additional pathological processes underlying DR but also offer a theoretical framework for identifying new therapeutic targets.

Mendelian Randomization (MR) is an approach that employs genetic variants as instrumental variables in order to evaluate the causal relationship between exposures and outcomes (17). In disease research, the MR approach effectively addresses confounding factors and reverse causation issues common in traditional observational studies (18). This study utilized MR to identify key genes related to mitochondrial function and PCD that were causally associated with DR, offering a novel perspective in unveiling the molecular mechanisms of DR.

In this investigation, transcriptomic data from the Gene Expression Omnibus (GEO) database related to DR were rigorously analyzed using bioinformatics methods. Through differential expression analysis, functional enrichment analysis, MR analysis, and PPI network construction, this study successfully identified a series of key genes related to DR. Furthermore, through gene set enrichment analysis (GSEA) and regulatory network analysis, this study elucidated the potential mechanisms of these key genes in DR and proposed several potential therapeutic targets. These findings offer new insights and methods for the diagnosis and treatment of DR, with the potential to improve treatment outcomes and quality of life for the afflicted individuals.

## 2 Materials and methods

### 2.1 Data source

The GEO database (https://www.ncbi.nlm.nih.gov/) was searched to acquire the transcriptome expression profile of the GSE94019 (GPL11154) and GSE60436 (GPL24120) datasets. The training set GSE94019 contained the retinal tissues of 13 DR patients and four normal people. The GSE60436 dataset comprised six DR retina samples and three control retina samples, serving as a validation set. The mitochondrial-related genes (MRGs) and PCD-related genes (PCDRGs) were acquired by assessing the pertinent literature (19). Furthermore, the outcome dataset of DR (ukb-b-12141) and the exposure factors datasets were obtained from the Integrative Epidemiology Unit (IEU) Open genome-wide association study (GWAS) database (https://gwas.mrcieu.ac.uk/). The ukb-b-12141 comprised 216,666 samples and 16,380,459 single nucleotide polymorphisms (SNPs). Finally, 680 apoptosis-related genes were obtained through literature search (20).

### 2.2 Differential expression analysis and functional annotation analysis

In the GSE94019 dataset, the limma package (v 3.52.4) (21) was utilized to identify the differentially expressed genes (DEGs) associated with DR. This was achieved via differential expression analysis between DR samples and control samples (|Log_2_FC| > 0.5, *p* < 0.05). Employing the ggplot2 package (v 3.3.6) (22) and the pheatmap package (v 0.4.9) (23), the volcano map and the heat map were constructed, respectively. Based on the GSE94019 dataset, Pearson correlation analysis was utilized to analyze the correlation between MRGs and PCDRGs. Genes with an absolute value of correlation greater than or equal to 0.8 and a *P* value lower than 0.05 were selected as both mitochondrial and PCD-related genes (M-PCD) for subsequent analysis. Then, utilizing the ggVennDiagram package (v 1.2.3) (24) in R, candidate genes were identified by overlapping DR-DEGs and M-PCD.

To delve deeper into the common function and enrichment pathways of candidate genes, functional annotation analysis was conducted using R package clusterProfile (v 4.4.4) (25) to explore the Gene Ontology (GO) functions, as well as Kyoto Encyclopedia of Genes and Genomes (KEGG) pathways (*p.*adj <0.05).

### 2.3 MR analysis

In MR studies, the following three presumptions need to be met: (a) instrumental variables (IVs) and exposure factors should be significantly correlated, (b) IVs should not be impacted by confounding variables connected to exposure factors or outcome, and (c) the impact of IVs on the outcome should be solely mediated by exposure factors. The candidate genes were utilized as exposure factors, while DR served as the outcome for two-sample MR analysis. Firstly, the unification of effect alleles and effect sizes was conducted by utilizing the harmonized data function in the TwoSampleMR R package (v 0.5.6) (26). Secondly, the extract instruments function was employed to select SNPs that displayed significant relationships with candidate genes (*p* < 5×10^-8^), which were then used as IVs. Meanwhile, the parameter clump was set as TRUE to eliminate the IVs with linkage disequilibrium (LD) (R^2^ = 0.001; kb=10000). Any IVs found to be significantly associated with the outcome were removed. Subsequently, the two-sample MR analysis was executed employing five methods [Weighted median (27), MR Egger (18), Inverse variance weighted (IVW) (28), Simple median (29), and Weighted mode (29)], with the primary method being IVW (*p* < 0.05).

The purpose of creating the scatter plot was to analyze the relationship between exposure factors and outcome. Meanwhile, the forest plot was utilized to evaluate how effective exposure factors were in predicting or diagnosing the outcome. Furthermore, the funnel plot was employed to investigate if IVs followed Mendel’s second law of random assortment.

The reliability of MR analysis was evaluated through sensitivity analysis. Firstly, Cochran’s Q test was utilized to assess heterogeneity. Genes with a *P* value of less than 0.05 indicated the presence of heterogeneity, suggesting that they should be excluded in subsequent analyses. Secondly, we conducted a horizontal pleiotropy test using the MR-Egger method. A *P*-value higher than 0.05 suggested that the influence of SNPs on outcomes was solely mediated by exposure factors. Lastly, the mr_leaveoneout function in TwoSampleMR was utilized to conduct a Leave-One-Out (LOO) analysis, aiming to investigate the potential impact of a single SNP on the overall effect. SNPs that, when removed, did not notably affect the outcomes were considered reliable. The candidate genes with a *P*-value from the IVW method less than 0.05 and validated by sensitivity analysis were identified as candidate key genes.

### 2.4 Establishment of PPI network and expression verification of key genes

The STRING database (https://string-db.org/) was utilized to predict the interactions among proteins corresponding to candidate key genes, with a confidence score threshold of 0.4. Cytoscape (v 3.8.2) (30) was utilized for visualizing the PPI network. Subsequently, node genes in the PPI network were selected for further analysis. By employing six algorithms (DEgree, DMNC, EPC, MCC, Genes are BottleNeck and MNC) within Cytoscape software (v 3.8.2), which evaluate and rank nodes genes based on their level of interaction, the common genes of the top 10 genes identified in these six algorithms were considered key genes. The expression of these key genes was analyzed in the GSE94019 dataset and the external validation dataset GSE60436.

### 2.5 Construction of gene-gene interactions network and GSEA analysis

In order to understand the interactions between key genes and other genes, as well as their associated biological functions, the GeneMANIA database (http://www.genemania.org/) was employed. This platform facilitated the prediction and construction of a GGI network. For a more thorough understanding of the functions and pathways involved in key genes, based on org.Hs.eg.db gene set, single gene GSEA analysis was conducted using clusterProfiler package (v 4.4.4) (25) in GSE94019 dataset (|NES| > 1, q < 0.25, *p*.adj < 0.05).

### 2.6 Immune infiltration analysis

The ssGSEA algorithm was employed to analyze the immune infiltration of the samples in the training set by determining the distribution proportion of 28 distinct types of immune cells within these samples. The difference in immune cell infiltration between DR and control groups was comparatively assessed by rank sum test (*P* < 0.05). The Spearman correlation analysis was employed to calculate the correlation between key genes and differential immune cells, and the R package ggcorrplot (v 0.1.4.1) (31) was utilized for visualizing the correlation.

### 2.7 The relationship between inflammatory cytokines and key genes

To elucidate the association between inflammatory cytokines and DR, this study investigated the correlation between inflammatory cytokines and key genes. Initially, a rank sum test based on the GSE94019 dataset was employed to assess the differential expression of inflammatory cytokines in control and DR groups (*P* < 0.01). The correlation between key genes and differential inflammatory cytokines was then examined. In addition, in the GSE94019 dataset, differences in the expression of apoptosis-related genes between DR samples and normal samples were examined using the Wilcoxon rank-sum test (P<0.05). The genes obtained were named the retinal vascular endothelial cell apoptosis-related genes, then the correlation between the key genes and genes related to differential apoptosis in retinal vascular endothelial cells was analyzed by spearman method. Among them, when the correlation value were all greater than 0.3, indicated correlation.

### 2.8 Construction of regulatory networks

The ChEA3 database (https://amp.pharm.mssm.edu/ChEA3) was utilized to search transcription factors (TFs) of the key genes, and the top 30 ranked TFs were selected for the construction of the TF-mRNA network.

The miRNAs that interact with key genes were predicted using the miRWalk (http://mirwalk.uni-hd.de/) and miRDB (http://mirdb.org) database. The miRNAs shared by the two databases were selected as key miRNAs. The starbase database (http://starbase.sysu.edu.cn/) was utilized for predicting the lncRNAs interacting with key miRNAs, and lncRNAs with clipExpNum greater than or equal to 20 were screened. The TF-mRNA networks and lncRNA-miRNA-mRNA networks were constructed using the Cytoscape software (v 3.8.2).

The target drugs of key genes were predicted in the Drug-Gene Interaction Database (DGIdb, http://www.dgidb.org) to establish a drug-gene network.

### 2.9 Animals model and experimental grouping

A total of 12 Sprague Dawley (SD) rats (six-week-old, 180 ± 20 g) were procured from Vital River Laboratory Animal Technology Co., Ltd (Grade SPF, SCXK (Zhejiang): SYXK2023-0003). These rats were housed in a controlled environment with specific pathogen-free conditions, maintaining a room temperature of around 23 ± 2°C, humidity levels at approximately 50 ± 10%, and a light/dark cycle of 12 hours each. The care of the animals was in accordance with institutional guidelines. The Laboratory Animal Management and Ethics Committee, Dongdian (Hangzhou) Medical Technology Co., LTD approved all animal-related protocols (EPI2023056), which adhered to the Regulations for the Administration of Affairs Concerning Experimental Animals sanctioned by the State Council of the People’s Republic of China. Random allocation led to the division of the rats equally into two groups: a control group and a DR group with five rats in each group.

Rats in the DR group were administered 30 mg/kg of streptozotocin (STZ) via intraperitoneal injection and fed high-fat chow for 14 consecutive weeks. After 72 hours of STZ injection, blood was collected from the tail vein of the rats to assess their fasting blood glucose concentration. If the concentration in the DR Group was ≥16.7mmol/L or significantly different from that of the normal group, it indicated the successful establishment of the DR model.

### 2.10 Validation of key genes by RT-qPCR

To validate the key genes identified through the analysis of the public database, five pairs of eyeball tissue samples were acquired from SD rats, comprising DR cases and controls. RNA isolation and RT-qPCR were performed on these ten tissue samples. The TRIzol method (Ambion, Austin, USA) was employed for the extraction of the total RNA following the provided instructions. Subsequently, the first-strand-cDNA-synthesis-kit (Servicebio, Wuhan, China) was employed for reverse transcription of total RNA into cDNA as per the provided protocol. qPCR analysis was then conducted using the 2xUniversal Blue SYBR Green qPCR Master Mix (Servicebio, Wuhan, China), following the provided protocol. The primers employed for PCR are listed in **Supplementary Table S1**. Gene expression levels were normalized to GAPDH, serving as an internal reference, and calculated using the 2−ΔΔCq method (32).

### 2.11 Immunohistochemistry validation of key genes

To further validate the key genes identified, immunohistochemistry was performed on retinal tissues from SD rats. Tissue sections were deparaffinized in xylene I and II for 5 minutes each, followed by rehydration in absolute ethanol, 95% ethanol, and 85% ethanol for 1 minute each. After washing with water, antigen retrieval was conducted using sodium citrate buffer (pH 6.0) heated in a microwave for 15 minutes. Sections were cooled and washed with water. Endogenous peroxidase was blocked by incubating the sections with 3% H2O2 at room temperature for 15 minutes, followed by three PBS washes. Sections were then blocked with goat serum at 37°C for 30 minutes. Primary antibodies against XCT and MYC were applied, and sections were incubated at 37°C for 1 hour. After washing, HRP-conjugated goat anti-rabbit IgG was applied as the secondary antibody and incubated at 37°C for 30 minutes. DAB chromogen was used for visualization, with sections incubated for 5 minutes at room temperature in the dark. The reaction was stopped with water, and the sections were observed under a microscope. Antibody information is provided in **Supplementary Table S2**.

### 2.12 Western blot validation of key genes

Western blotting was performed to assess the protein expression of key genes in retinal tissues from SD rats. Tissues were washed 1-2 times with pre-chilled PBS, cut into small pieces, and lysed using a homogenizer with 10 volumes of lysis buffer. The lysates were incubated on ice or at 4°C for 30 minutes, followed by centrifugation at 12,000 rpm for 10 minutes at 4°C. The supernatant was collected for total protein analysis. Protein concentration was determined using the BCA protein assay kit according to the manufacturer’s instructions. For denaturation, protein samples were mixed with 5x reducing sample buffer at a 4:1 ratio and heated at 95°C for 10 minutes. Proteins were separated by SDS-PAGE at 200V for 30 minutes and transferred to PVDF membranes, activated in ethanol for 2 minutes, and transferred at 300mA for 30 minutes. Membranes were blocked with 5% skim milk at room temperature for 30 minutes, followed by overnight incubation with diluted XCT and MYC primary antibodies at 4°C. After washing, membranes were incubated with secondary antibodies at room temperature for 30 minutes, followed by three washes. Protein bands were detected using ECL chemiluminescence, and images were saved as TIFF files. Grayscale analysis was performed using AIWBwellTM software. Antibody information is listed in **Supplementary Table S3**.

### 2.13 Statistical analysis

The R software (v 4.2.1) was utilized to process and analyze the data. The *P-*value < 0.05 was deemed statistically significant.

## 3 Results

### 3.1 Acquisition of 658 candidate genes

**Figure 1:**
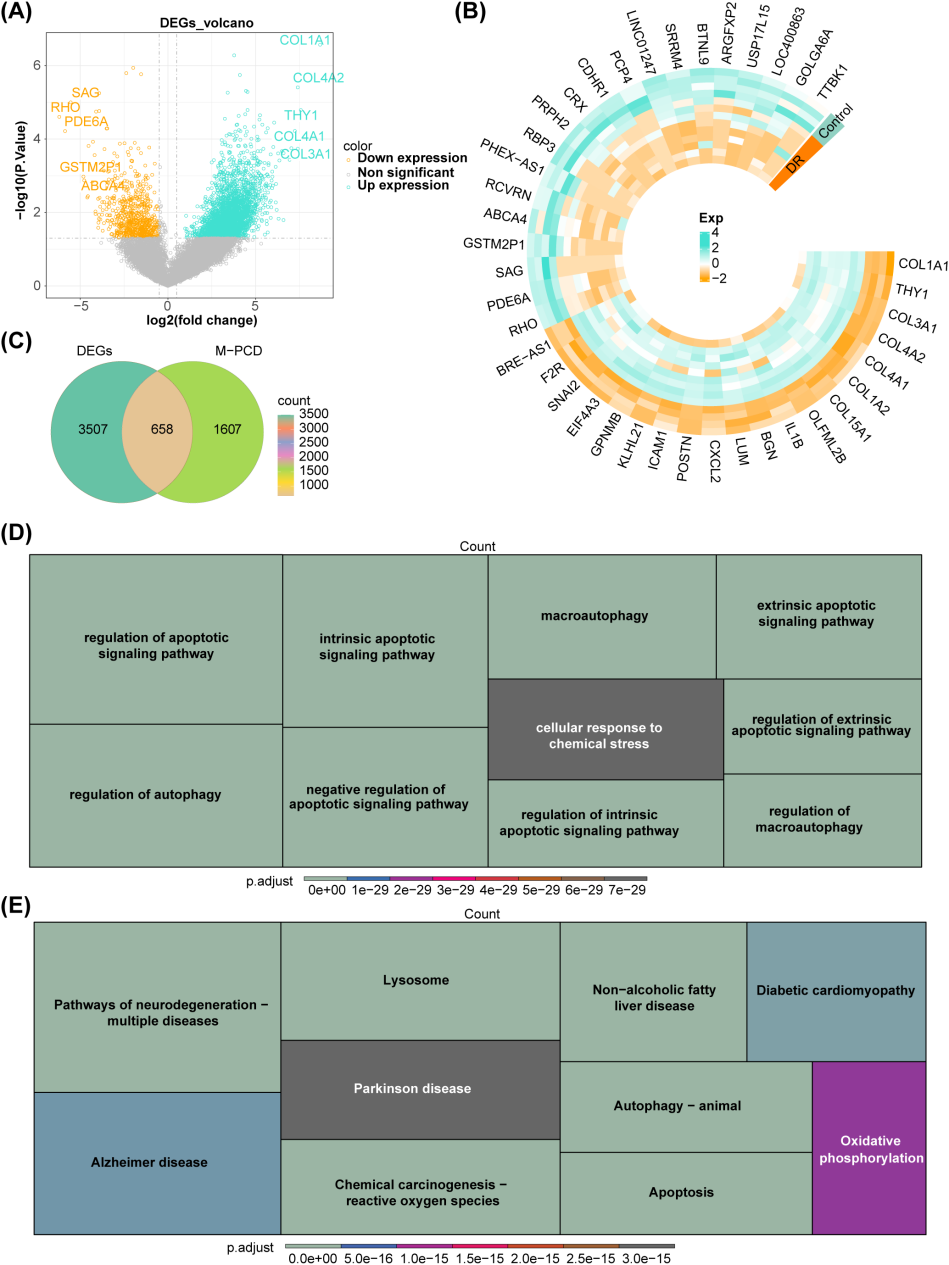
Acquisition and functional enrichment analysis of candidate genes **(A)** Volcano map of DR-DEGs **(B)** Heat map of DR-DEGs **(C)** Venn map of candidate genes **(D)** GO functions enriched by candidate genes **(E)** KEGG pathways enriched by candidate genes.

A total of 4,165 DR-DEGs were determined in DR samples relative to control samples, comprising 3,346 upregulated and 819 downregulated DR-DEGs. The volcano map displayed these DR-DEGs and labeled the top five upregulated and downregulated DR-DEGs sequenced by log_2_FC (**Figure 1A**). Additionally, the heat map showed the expression of some DR-DEGs between DR group and control group (**Figure 1B**). The correlation analysis between MRGs and PCDRGs displayed that there were 2,265 M-PCD with absolute correlations values greater than or equal to 0.8 and *P* values less than 0.05. The intersection of DR-DEGs and M-PCD was utilized to obtain 658 candidate genes (**Figure 1C**).

The 658 candidate genes were enriched in 2,272 GO functions, encompassing intrinsic apoptotic signaling pathway, regulation of apoptotic signaling pathway, mitochondrial inner membrane, ubiquitin-like protein ligase binding, etc. (**Figure 1D**). A total of 151 KEGG pathways exhibited significant enrichment, including pathways such as lysosome, non-alcoholic fatty liver disease, pathways of neurodegeneration - multiple diseases, autophagy - animal and apoptosis (**Figure 1E**).

### 3.2 Identification and validation of key genes

**Figure 2:**
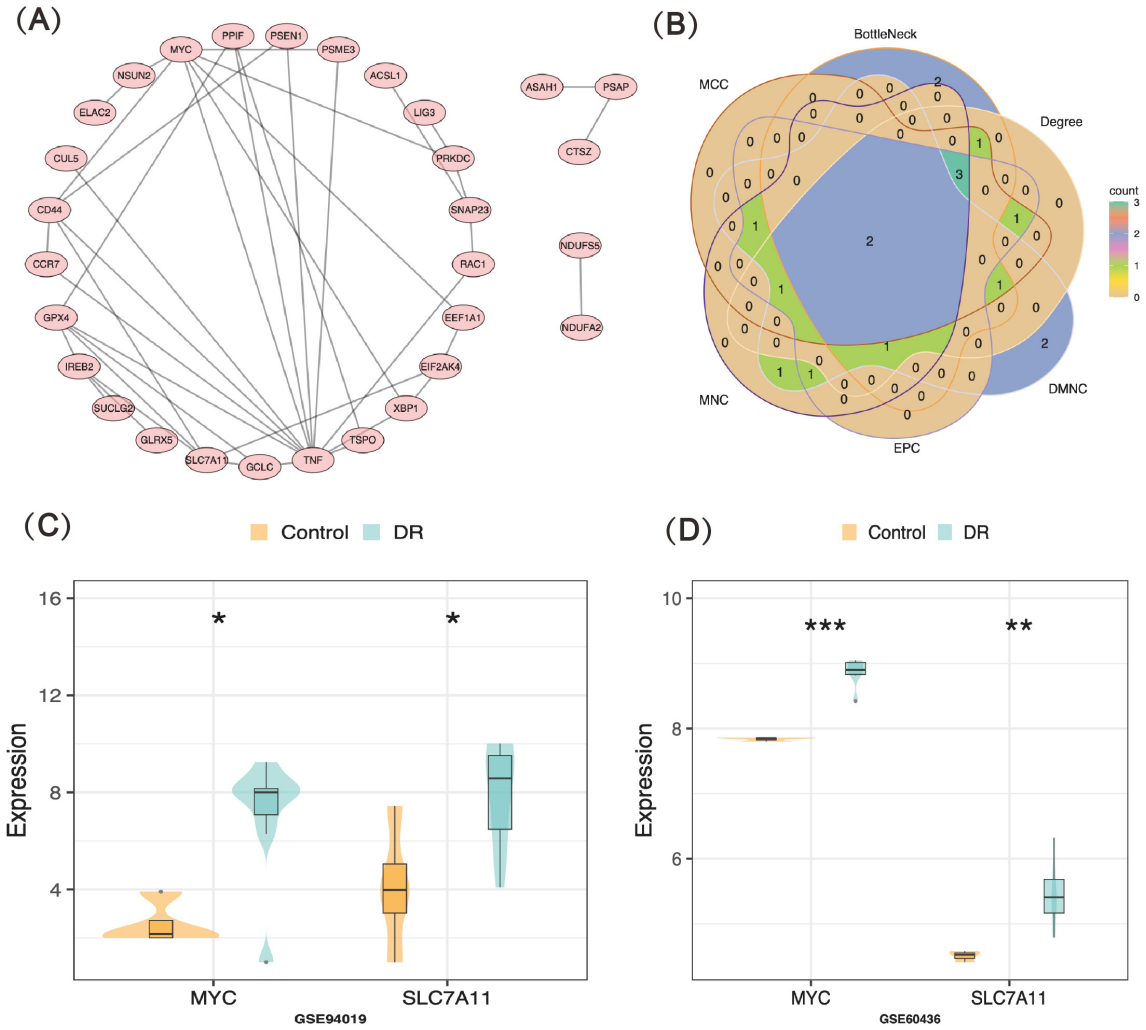
Identification and validation of key genes **(A)** PPI network of candidate genes **(B)** Venn map of key genes (MYC and SLC7A11) **(C, D)** The expression levels of MYC and SLC7A11 in GSE94019 and GSE60436 datasets. *: p < 0.05; **: p < 0.01; ***: p < 0.001.

Forty-three candidate key genes demonstrated causal relationships with DR (*P*<0.05 in IVW). Among the 43 candidate key genes, 13 did not interact with other genes. The remaining 30 candidate key genes were used to construct the PPI network and served as node genes for subsequent analysis (**Figure 2A**). The top ten genes were selected for intersection based on their rankings in the DEgree, DMNC, EPC, MCC, BottleNeck, and MNC algorithms (**Figure 2B**, **Supplementary Figure S1**). Consequently, two key genes (MYC and SLC7A11) were identified. Expression analysis results also confirmed the consistent expression trend of the two key genes in both the GSE94019 and GSE60436 datasets, revealing a significant increase in the DR group compared to control group (**Figures 2C, D**). The results of the correlation analysis of the key genes (MYC and SLC7A11) with 168 retinal vascular endothelial cell apoptosis-related genes showed that the correlation values of MYC and SLC7A11 with the retinal vascular endothelial cell apoptosis-related genes were all greater than 0.4, which indicated that MYC and SLC7A11 were significantly correlated with retinal vascular endothelial cell apoptosis-related genes (**Supplementary Table S4**).

### 3.3 MR analysis of MYC and SLC7A11

**Figure 3:**
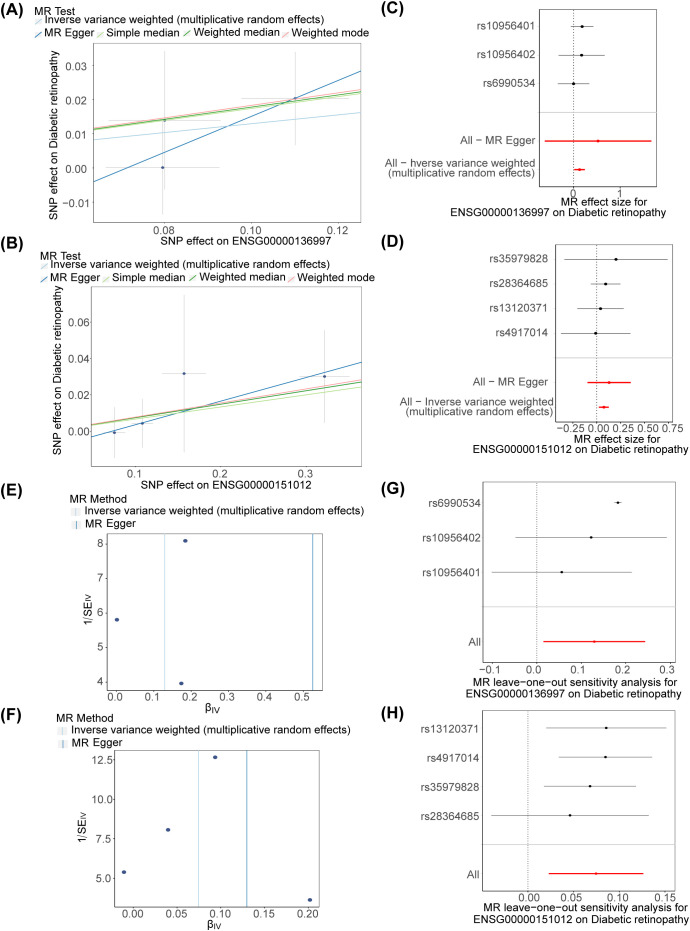
MR analysis of key genes **(A, B)** Scatter plots of MYC and SLC7A11 **(C, D)** Forest maps of MYC and SLC7A11 **(E, F)** Funnel plots of MYC and SLC7A11 **(G, H)** Forest maps of MYC and SLC7A11 in LOO analysis.

The odd ratios (OR) of MYC and SLC7A11 in IVW method were greater than 1, suggesting that these two genes were risk factors for DR (**Table 1**). This finding was further supported by the scatter plot, which revealed an overall positive correlation between the effect of SNPs of these two genes and DR (**Figures 3A, B**). Moreover, the forest map illustrated that MYC and SLC7A11 were risk factors, with the MR effect size exceeding 0 (**Figures 3C, D**). The funnel plot showed that MR analysis of candidate key genes and DR was consistent with Mendel’s second random law (**Figures 3E, F**).

**Table 1:** MR analysis results.

outcome	exposure	Method	Pvalue	OR	OR_lci95	OR_uci95
DR	MYC	MR Egger	0.533	1.692	0.539	5.304
		Inverse variance weighted (multiplicative random effects)	0.027	1.138	1.015	1.276
		Weighted median	0.081	1.195	0.978	1.459
		Weighted mode	0.287	1.201	0.936	1.54
		Simple median	0.186	1.19	0.919	1.539
	SLC7A11	MR Egger	0.379	1.139	0.907	1.429
		Inverse variance weighted (multiplicative random effects)	0.005	1.077	1.023	1.135
		Weighted median	0.273	1.077	0.943	1.23
		Weighted mode	0.333	1.081	0.947	1.233
		Simple median	0.407	1.069	0.913	1.252

The heterogeneity test revealed no evidence of heterogeneity for MYC and SLC7A11 ( *P* > 0.5) (**Table 2**). Additionally, the *P*-values obtained from the horizontal pleiotropy test for MYC and SLC7A11 all exceeded 0.05, indicating an absence of horizontal pleiotropy. (**Table 3**). The LOO analysis showed that removing each SNP did not significantly change the impact of the remaining SNP on the outcome. The findings of sensitivity analysis provided evidence for the reliability of our MR analysis results (**Figures 3G, H**).

**Table 2:** The result of heterogeneity test.

outcome	exposure	Q	Q_df	Q_pval
DR	MYC	0.783	2	0.676
	**SLC7A11**	0.56	3	0.905

**Table 3:** The result of horizontal pleiotropy test.

outcome	exposure	egger_intercept	se	pval
DR	MYC	-0.037	0.054	0.616
	**SLC7A11**	-0.009	0.017	0.631

### 3.4 Enrichment of the key genes in diabetes and cancer-related pathways

**Figure 4:**
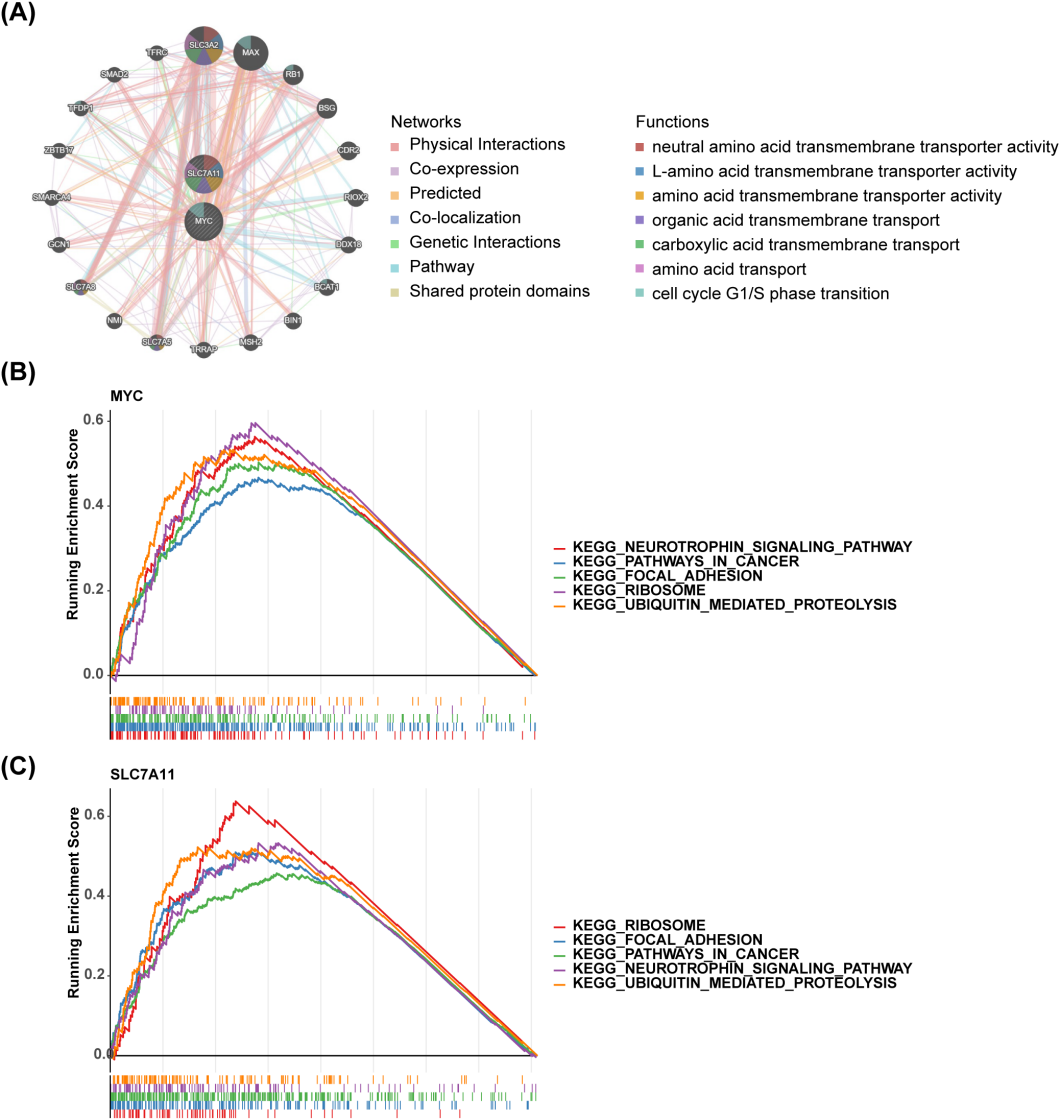
Functional enrichment analysis of key genes **(A)** GeneMINIA network of MYC and SLC7A11 **(B, C)** GSEA analysis of MYC and SLC7A11.

In the GeneMINIA network, 20 genes were found to interact with key genes, such as SLC3A2, MAX, RB1, SLC7A5, and TFRC. The biological functions related to SLC7A11 included L-amino acid transmembrane transporter activity, amino acid transmembrane transporter activity, neutral amino acid transmembrane transporter activity, organic acid transmembrane transport, carboxylic acid transmembrane transport, and amino acid transport. The cell cycle G1/S phase transition was associated with MYC (**Figure 4A**). MYC significantly enriched 91 pathways, with SLC7A11 being enriched in 80 KEGG pathways (**Figures 4B, C**). The pathways in which both genes were enriched encompassed the neurotrophin signaling pathway, pathways in cancer, focal adhesion, ribosome, ubiquitin-mediated proteolysis, etc.

### 3.5 Differential immune infiltration in the DR group compared to the control group

**Figure 5:**
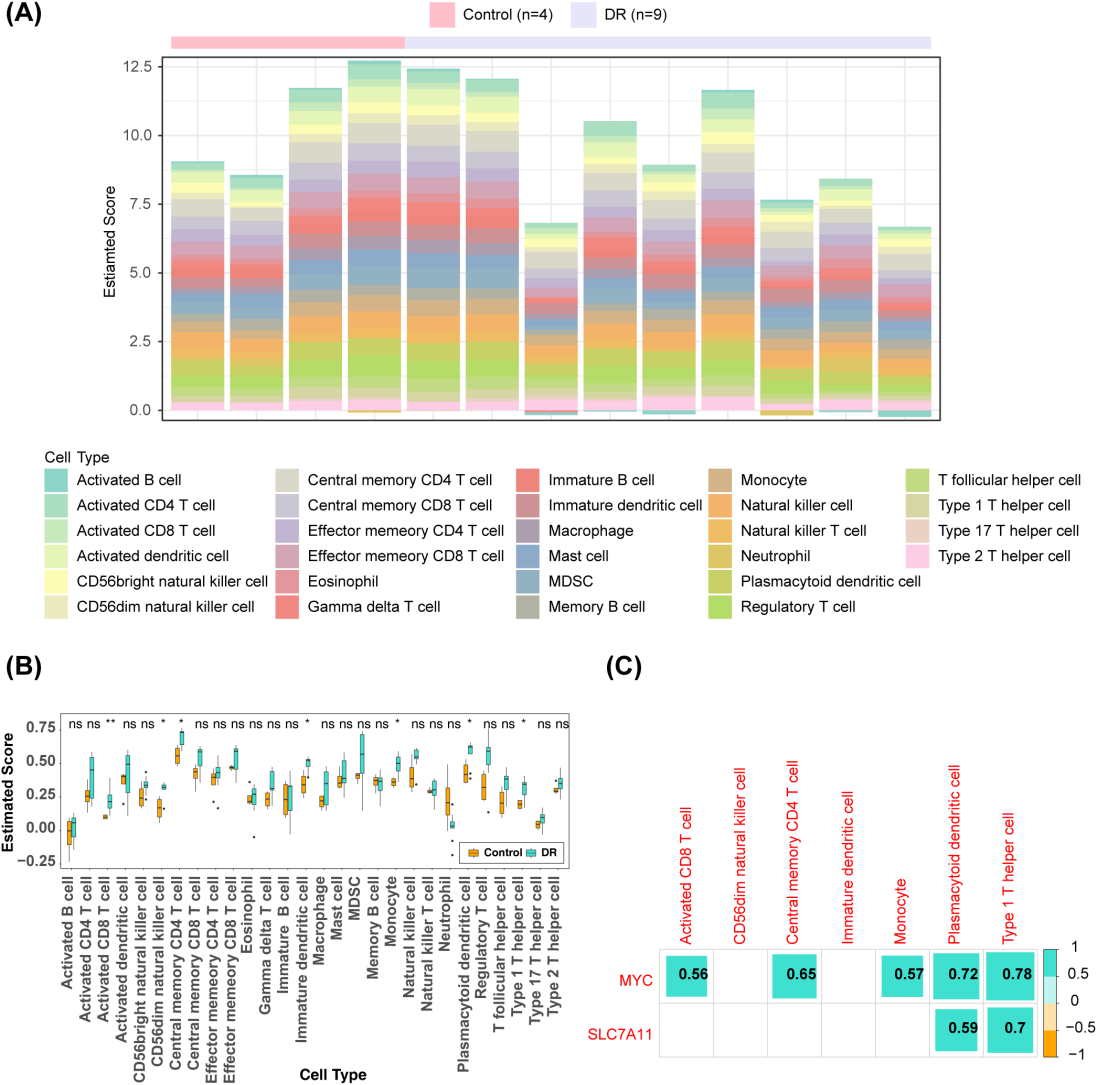
Immune infiltration analysis **(A)** Heat map of the distributions of the 28 immune cells **(B)** Differences in the abundance of immune cells in DR and Control groups **(C)** Differential immune cell and key genes correlation heat map (the number represents the correlation coefficient). *: p < 0.05; **: p < 0.01; ns, not significant.

The heat map showed the proportion of 28 types of immune cell infiltration in the DR group and the control group (**Figure 5A**). The groups exhibited significant differences in seven immune cell abundance scores, comprising activated CD8^+^ T cells, CD56^dim^ natural killer (NK) cells, central memory CD4^+^ T cells, immature dendritic cells, monocytes, plasmacytoid dendritic cells, and type 1 helper T cells (**Figure 5B**). The expression of SLC7A11 exhibited a notably positive correlation with the abundance of plasmacytoid dendritic cells (R = 0.59) and type 1 T helper cells (R = 0.7). The activated CD8^+^ T cells (R = 0.56), central memory CD4^+^ T cells (R = 0.65), monocytes (R = 0.57), plasmacytoid dendritic cells (R = 0.72), and type 1 T helper cells (R = 0.78) showed a markedly positive correlation with the expression of MYC (**Figure 5C**).

### 3.6 Significant positive correlation between key genes and most inflammatory cytokines

**Figure 6:**
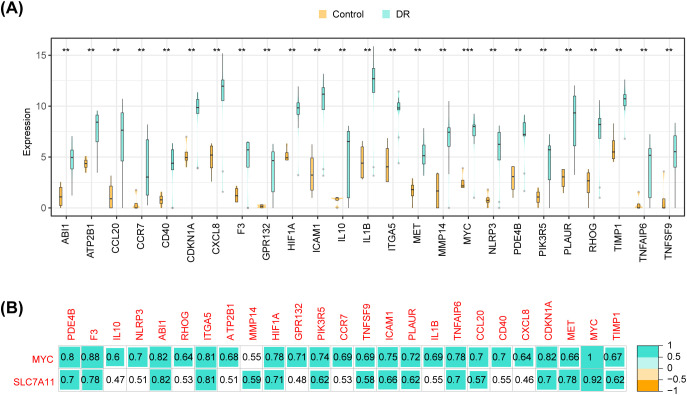
Inflammatory cytokines analysis **(A)** Differences in expression of inflammatory cytokines in DR and Control groups **(B)** Correlation heat map of differential immune cytokines and key genes (the number represents the correlation coefficient).

There were 25 inflammatory cytokines, such as ABI1, ATP2B1, CCL20, and CCR7, with significant differences in expression between the DR group and the control group (**Figure 6A**). Correlation analysis showed that MYC and SLC7A11 exhibited positive correlation with most of the differential immune cytokines (**Figure 6B**). Specifically, MYC exhibited the highest positive correlation with F3 (R = 0.88), while SLC7A11 demonstrated the strongest association with MYC (R = 0.92).

### 3.7 Potential roles of hsa-miR-206, hsa-miR-26a-5p, and hsa-miR-3129-5p in DR

**Figure 7:**
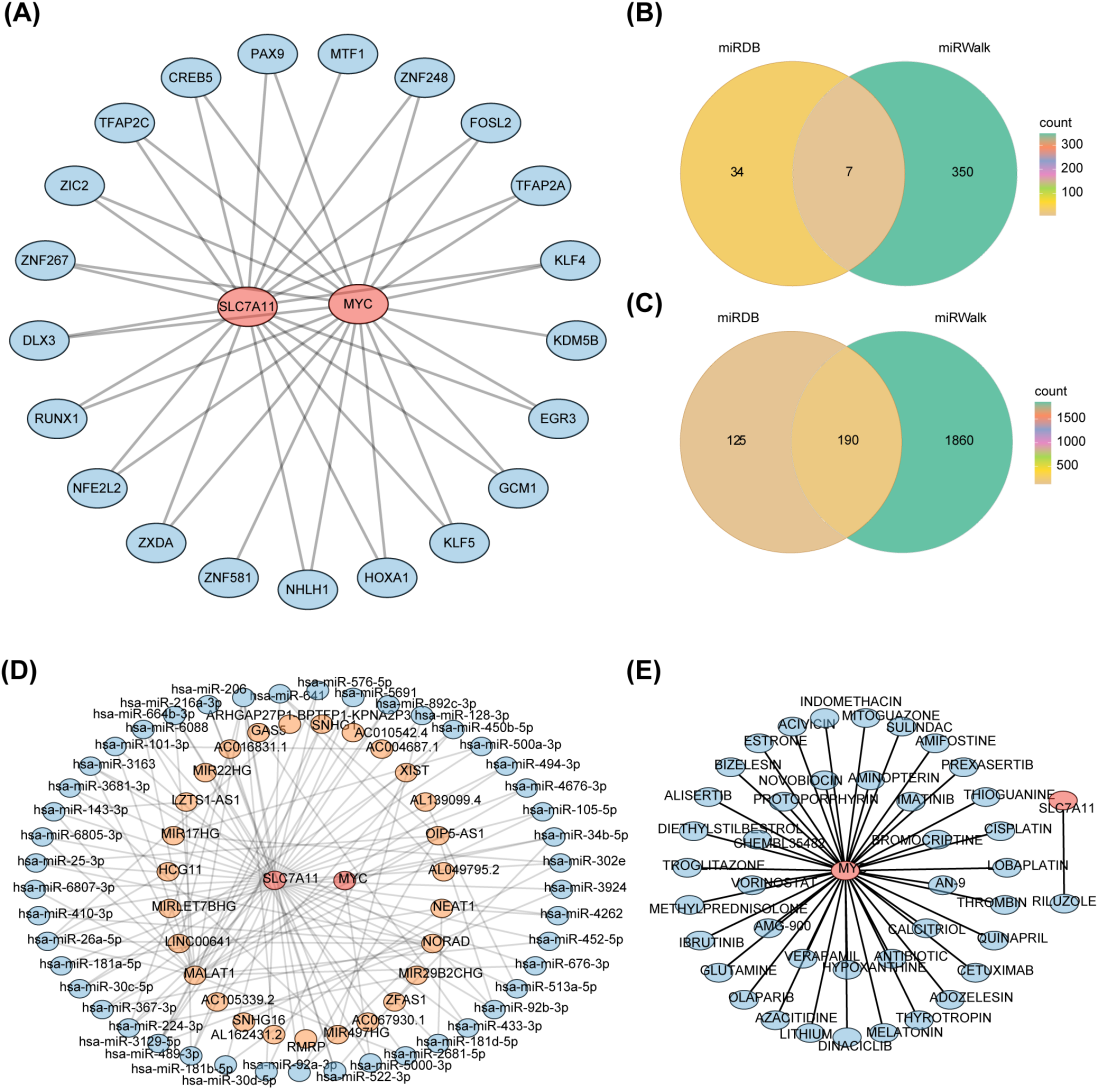
Construction of regulatory networks **(A)** TFs-mRNA network (Blue represents TF, and red represents key genes) **(B)** Venn map of seven key miRNAs associated with MYC **(C)** Venn map of 190 key miRNAs associated with SLC7A11 **(D)** LncRNA-miRNA-mRNA network (Blue represents miRNAs, orange represents lncRNAs, and red represents key genes) **(E)** Drug-mRNA network (Blue represents drugs, and red represents key genes).

The TFs-mRNA network consisted of 23 nodes and 39 edges (**Figure 7A**). Within this network, 18 TFs were simultaneously predicted by both MYC and SLC7A11, including GCM1, FOSL2, DLX3, EGR3, CREB5, etc.

The MYC was predicted to be associated with seven key miRNAs, while SLC7A11 was associated with 190 key miRNAs (**Figures 7B, C**). Ultimately, the lncRNA-miRNA-mRNA network was established, consisting of two key genes, 27 lncRNAs and 45 miRNAs (**Figure 7D**). Four lncRNAs (RMRP, MALAT1, LINC00641, AL162431.2) and SLC7A11 competitively interacted with hsa-miR-206. SLC7A11, NORAD, HCG11, ARHGAP27P1-BPTFP1-KPNA2P3, MALAT1 were associated with hsa-miR-26a-5p. On the other hand, SLC7A11, AC010542.4, MALAT1, SNHG1, and NORAD were linked with hsa-miR-3129-5p. These three miRNAs exhibited the highest number of TFs and genes linked, suggesting their potential significance in DR.

In the DGIdb databases, a total of 40 target drugs, such as glutamine, AN-9, protoporphyrin, hypoxanthine, and addozoline, were predicted by MYC. Riluzole was identified as the target drug for SLC7A11 (**Figure 7E**).

### 3.8 *In vivo* validation of MYC and SLC7A11

**Figure 8:**
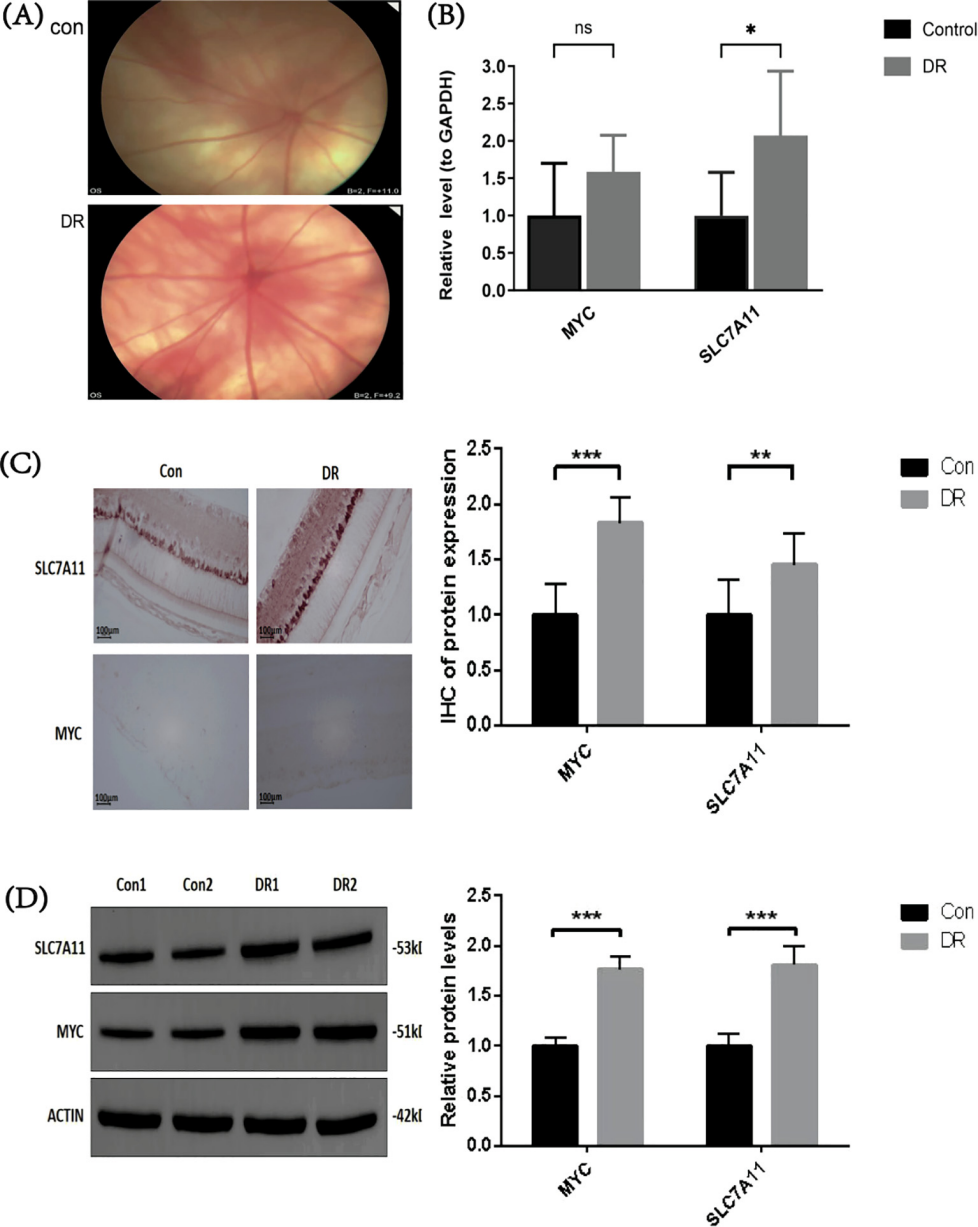
Expression of MYC and SLC7A11 in Control and DR Rats Models **(A)** Fundus images of control and DR rats. **(B)** Comparison of RT-PCR levels between control and DR rats (n = 3). **(C)** RT-PCR levels of MYC and SLC7A11 in control and DR rats (n = 3). **(D)** RT-PCR levels of MYC and SLC7A11 in control and DR rats (n = 3). Data are presented as mean ± SD (*p < 0.05, **p < 0.01, ***p < 0.001, ns, not significant, p > 0.05).

To assess and validate the expression of MYC and SLC7A11 in DR, retinal tissues were collected from both control and DR groups. **Figure 8A** shows the fundus images of rats in both groups. We compared the expression of MYC and SLC7A11 in the retinal tissues of control and DR rats. RT-PCR analysis indicated that while MYC expression was higher in the DR group compared to the control group, the difference was not statistically significant. However, SLC7A11 expression was significantly elevated in the DR group (**Figure 8B**). IHC analysis showed that both MYC and SLC7A11 were significantly upregulated in the DR group compared to the control group, with statistical significance between the groups (**Figure 8C**). Similarly, Western blot (WB) analysis demonstrated that the expression levels of MYC and SLC7A11 were significantly higher in the DR group compared to the control group (**Figure 8D**). Overall, these findings indicate that MYC and SLC7A11 are significantly upregulated in DR rat models.

## 4 Discussion

DR is a severe ocular complication of diabetes. Primarily, it manifests as a set of characteristic pathological alterations arising from diabetes-induced damage to retinal microvasculature, potentially resulting in blindness in severe instances. Despite advancements in elucidating the pathogenesis of DR, its treatment remains a primary focus of current ophthalmic disease research. Mounting evidence suggests that inflammation, oxidative stress, apoptosis, and autophagy within the Müller cells of the retina are intricately associated with DR (33). DR is one of the five major pathways of the most serious microvascular complications caused by hyperglycemia. These pathways include polyols, hexosamine, protein kinase C, angiotensin II pathways, and the accumulation of advanced glycation end products. The heightened production of reactive oxygen species (ROS) caused by hyperglycemia leads to local inflammation, mitochondrial dysfunction, microvascular dysfunction, and cell apoptosis. The accumulation of ROS, local inflammation, and cell death are closely related and significantly affect various stages of DR pathogenesis. Mitochondrial autophagy, a conserved multi-step pathway, plays a pivotal role in selectively degrading and restoring damaged mitochondria. In addition, microvascular dysfunction can cause ischemia and local inflammation, culminating in neovascularization, macular edema, and neurological dysfunction, ultimately resulting in long-term blindness (34). The link between TRX/TXNIP and redox signaling pathways encompasses the activation of Nod-like receptor thermal protein domain protein 3 (NLRP3) inflammasomes, cell apoptosis, autophagy/mitochondrial autophagy, and epigenetic modifications in redox-dependent pathways (35). DR promotes the formation of ER mitochondrial coupling and accelerates Ca^2+-^dependent cell apoptosis through the IP3R1-GRP75-VDAC1 axis (36). Research has highlighted that circulating mitochondrial DNA (mtDNA) levels are linked to DR, and alterations in mtDNA induced by hyperglycemia in early diabetes may be critically involved in the inflammation and progression of DR (37). In conclusion, mitochondria and apoptosis play pivotal roles in the occurrence of DR. To further comprehend their mechanisms in DR, two key genes (SLC7A11 and MYC) were identified through bioinformatics analysis in this study.

Studies have shown that SLC7A11 expression is suppressed under hyperglycemic conditions, leading to decreased glutathione (GSH) synthesis and reduced GPX4 activity, which ultimately results in increased lipid peroxidation and damage to membrane integrity (34). Thus, SLC7A11 may serve as an important biomarker for DR. P53 induces ferroptosis by inhibiting SLC7A11 or enhancing SAT1 expression, and SLC7A11 is enriched in the P53 signaling pathway, suggesting its potential role in DR pathogenesis through this pathway (35).

c-MYC, a pivotal member of the MYC gene family, is closely linked to angiogenesis and serves as a key driver of DR progression. c-Myc not only promotes endothelial cell angiogenesis but also enhances the proliferation of rat retinal pigment epithelial cells. Studies indicate that c-myc knockout reduces the release of pro-inflammatory factors from Müller cells, thereby slowing DR progression (36). Furthermore, the compound 221S-1a inhibits the G1/S phase transition by blocking the ERK1/2-c-Myc pathway, thereby reducing angiogenesis in both tumors and oxygen-induced retinopathy (37). c-Myc regulates endothelial cell proliferation and migration by selectively splicing MKNK2, a downstream molecule of the ERK signaling pathway; inhibition of MKNK2 enhances the migration of human umbilical vein endothelial cells (38). MYC also influences DUSP5 transcription via the ERK signaling pathway (39). Additionally, studies have found that ERBB3 modulates MYC activation by regulating AKT2 phosphorylation, thereby affecting the proliferation and migration of high-glucose-injured HUVECs (40).

Our study found that MYC expression is significantly upregulated in DR patients and is enriched in the MAPK/ERK signaling pathway. Based on this, we hypothesize that reducing MYC expression may alleviate DR progression by modulating the MAPK/ERK pathway. Furthermore, MYC and SLC7A11 are significantly enriched in pathways such as neurotrophin signaling, focal adhesion, and ubiquitin-mediated proteolysis, which are closely associated with retinal pathology in DR patients (42).

It has also been shown that inflammatory molecules in the retina are produced not only by leukocytes but also by glial cells. The retina’s inability to adapt to metabolic stress leads to glucose-mediated microvascular damage and chronic inflammation, ultimately resulting in retinal neurodegeneration and functional impairment. As a key amino acid transporter, SLC7A11’s reduced activity is closely associated with decreased glutathione (GSH) levels, leading to increased oxidative stress in retinal cells and accelerating retinal degeneration (38). As a transcription factor, MYC regulates the cell cycle, metabolism, and apoptosis, disrupting retinal structure and function and indirectly affecting photoreceptor response and neural signal transmission, further promoting retinal degeneration (39–41).

Our research also indicates that SLC7A11 and MYC are significantly associated with genes involved in retinal vascular endothelial cell apoptosis, suggesting that MYC may influence endothelial cell survival by regulating the expression of these genes. In the context of diabetic retinopathy, MYC upregulation has been associated with increased expression of apoptotic genes and accelerated endothelial cell apoptosis (42). SLC7A11 is similarly associated with increased oxidative stress and accelerated apoptosis (38). Therefore, SLC7A11 and MYC may directly or indirectly promote retinal degeneration by affecting metabolism, antioxidant defense, cell proliferation, and apoptosis.

Additionally, MiR-195 enhances EMT and cell permeability in high glucose-stimulated ARPE-19 cells by upregulating VEGFA/Snail1 and inhibiting Smurf2-mediated YY1 ubiquitination (43). Anti-VEGF therapy treats retinal degeneration by inhibiting neovascularization and controlling related pathological processes. Changes in the microenvironment during treatment may indirectly affect the expression or activity of MYC and SLC7A11. Although evidence suggests that metabolic conditions may indirectly influence MYC expression or function (5), its direct role has not been fully confirmed and requires further investigation. SLC7A11 may reduce vascular leakage and permeability, thereby lowering oxidative stress in retinal cells and reducing the demand for cystine uptake mediated by SLC7A11 (44). Furthermore, the natural diterpenoid EKO alleviates DR by maintaining vascular endothelial integrity through the c-fos/focal adhesion axis and activating the deubiquitinase ATXN3 (43).

Additionally, natural diterpenoid EKO activates deubiquitinase ATXN3 to maintain vascular endothelial integrity and alleviate DR via the c-fos/focal adhesion axis (45).

As a pro-inflammatory mediator, low-grade inflammation can trigger various cellular abnormalities and tissue damage, ultimately affecting the retina. This process involves increased levels of adhesion molecules, chemokines, and growth factors, which contribute extensively to the pathogenesis of DR. Inflammatory cells within the retina also respond to injury and stress. Moreover, inflammation exacerbates retinal neurodegeneration during the early stages of DR. Chronic inflammation plays a pivotal role in the progression of DR, particularly during its early stages (46). Hyperglycemia exacerbates oxidative stress by elevating the production and activation of advanced glycation endproduct(AGE), protein kinase c(PKC), and the flux of the polyol and hexosamine pathways. Enhanced oxidative stress, coupled with reduced levels of GSH, leads to the production of inflammatory intermediates Notably, arachidonic acid metabolites serve as significant inflammatory intermediates. Consequently, this cascade of events contributes to BRB rupture and heightened vascular permeability in the retina (47). Research indicates that individuals with prediabetes exhibit elevated levels of multiple inflammatory markers, including resistin, interleukin-6 (IL-6), TNF-α, interleukin-1β (IL-1β), and monocyte chemoattractant protein-1 (MCP-1), in their serum along with high fasting blood glucose levels. Furthermore, elevated levels of chemokines, including MCP-1, CCL2, and CCL5, and pro-inflammatory cytokines, such as TNF-α, IL-1β, and IL-6, have been reported (48). Chronic inflammation is characterized by the accumulation of macrophages, lymphocytes, and mature B cells within the affected tissues. These white blood cells extravasate from the blood vessels and gradually accumulate in the tissues due to the long-term release of inflammatory factors (49). The pathophysiology of DR is intricate. Elevated intracellular glucose levels in diabetic patients trigger the polyol pathway, resulting in the metabolism of glucose (49). This process results in AGE deposition, activation of PKC, and upregulation of AGE receptors and hexokinase pathways (50). Moreover, it triggers oxidative stress, leading to an increase in intracellular ROS and irreparable cell damage. Summarizing the above literature results, previous investigations have found that the occurrence of DR is intricately linked to inflammation. Likewise, the current study observed elevated expression of numerous inflammatory factors in patients with DR. Moreover, MYC and SLC7A11 showed significant positive correlations with these inflammatory markers. These findings suggest that MYC and SLC7A11 may induce an inflammatory response by upregulating the expression of inflammatory factors, thereby contributing to the development of DR.

Research has shown that Steroid Receptor Coactivator 2 (SRC-2), as a key mediator of steroid signaling, can stimulate the upregulation of the c-Myc-mediated amino acid transporter Slc7a5, thereby controlling the activation of CD4+ T cells (1). This mechanism suggests that steroids can indirectly regulate c-Myc activity through SRC coactivators, with c-Myc acting as a core transcription factor playing a critical role in cell growth, metabolism, and apoptosis. SRC-2 has been shown to mediate the suppression of MYC. On the other hand, studies have indicated that SRC-2 can coactivate anti-tumor target genes, thereby inhibiting MYC-induced liver cancer progression (51). This implies that in the steroid signaling pathway, SRC coactivators may exert opposing regulatory effects on MYC depending on the biological context. Although current research on the effects of steroids on SLC7A11 is limited, considering the critical role of SLC7A11 in redox balance and ferroptosis regulation, we hypothesize that steroids might influence SLC7A11 expression or function through their indirect regulation of MYC activity.

Xiaoli Xiang et al. observed a higher presence of CD4+ cells in patients with DR (52). In this study, the infiltration proportion of central memory CD4+ T cells in the DR group also exhibited a significant increase. Additionally, MYC showed a significant positive correlation with central memory CD4+ T cells. These findings suggest a potential role for MYC in the occurrence of DR by regulating the proportion of central memory CD4+ T cells in affected individuals.

In this study, we found that mitochondria and PCD-related genes MYC and SLC7A11 play important roles in DR. And previous studies have shown that regulating the activities of MYC and SLC7A11 can enhance the resistance of retinal cells to oxidative stress, reduce cell death, and ultimately protect vision (44). In view of this, it is reasonable to hypothesize that MYC and SLC7A11 are not only the key to understanding the pathological mechanisms of DR, but are also expected to be potential targets for the diagnosis and treatment of DR, which will bring a new diagnostic and treatment strategy for DR patients.

We performed bioinformatic analysis using public database data, which revealed the potential roles of MYC and SLC7A11 in diabetic retinopathy (DR). Subsequently, *in vivo* experiments were conducted to further validate the elevated expression of these two genes in DR. Results from RT-qPCR, immunohistochemistry, and western blot analyses consistently showed that the expression levels of MYC and SLC7A11 were significantly higher in the retinal tissues of DR rats compared to controls. These findings align with the bioinformatic analysis, suggesting that MYC and SLC7A11 play crucial roles in DR pathogenesis by regulating oxidative stress, apoptosis, and mitochondrial dysfunction. Thus, modulating the expression of MYC and SLC7A11 may present new therapeutic targets for DR. Further studies are needed to explore the molecular mechanisms of these genes and their potential as therapeutic targets. Despite these important findings, the study has some limitations. For key regulatory genes like MYC and SLC7A11, changes in their expression could significantly impact disease progression. However, due to funding and experimental constraints, we were unable to conduct further *in vitro* experiments, including gene overexpression or silencing validation.

In future research, we plan to validate these findings using DR patient samples. We will combine gene silencing or overexpression experiments, utilizing siRNA or gene transfection techniques to manipulate MYC and SLC7A11 expression in cell or animal models. This approach will allow us to observe their effects on mitochondrial function and cell apoptosis, providing a more comprehensive understanding of the roles of MYC and SLC7A11 in diabetic retinopathy and offering scientific evidence for disease prevention and treatment.

## Data Availability

The datasets presented in this study can be found in online repositories. The names of the repository/repositories and accession number(s) can be found in the article/**Supplementary Material**.

## Supplementary material

Supplementary Table 1 The primers employed for PCR.

Supplementary Table 2 Antibody Information for IHC.

Supplementary Table 3 Antibody Information for WB.

Supplementary Table 4 Results of correlation analysis between key genes and differential apoptosis genes.
