# Supplementary figures and images for: Transcriptome combined with Mendelian randomization to screen key genes associated with mitochondrial and programmed cell death causally associated with diabetic retinopathy
Source: Front Endocrinol (Lausanne). 2024 Nov 20;15:1422787. doi: 10.3389/fendo.2024.1422787 (PMC11615439; doi:10.3389/fendo.2024.1422787)

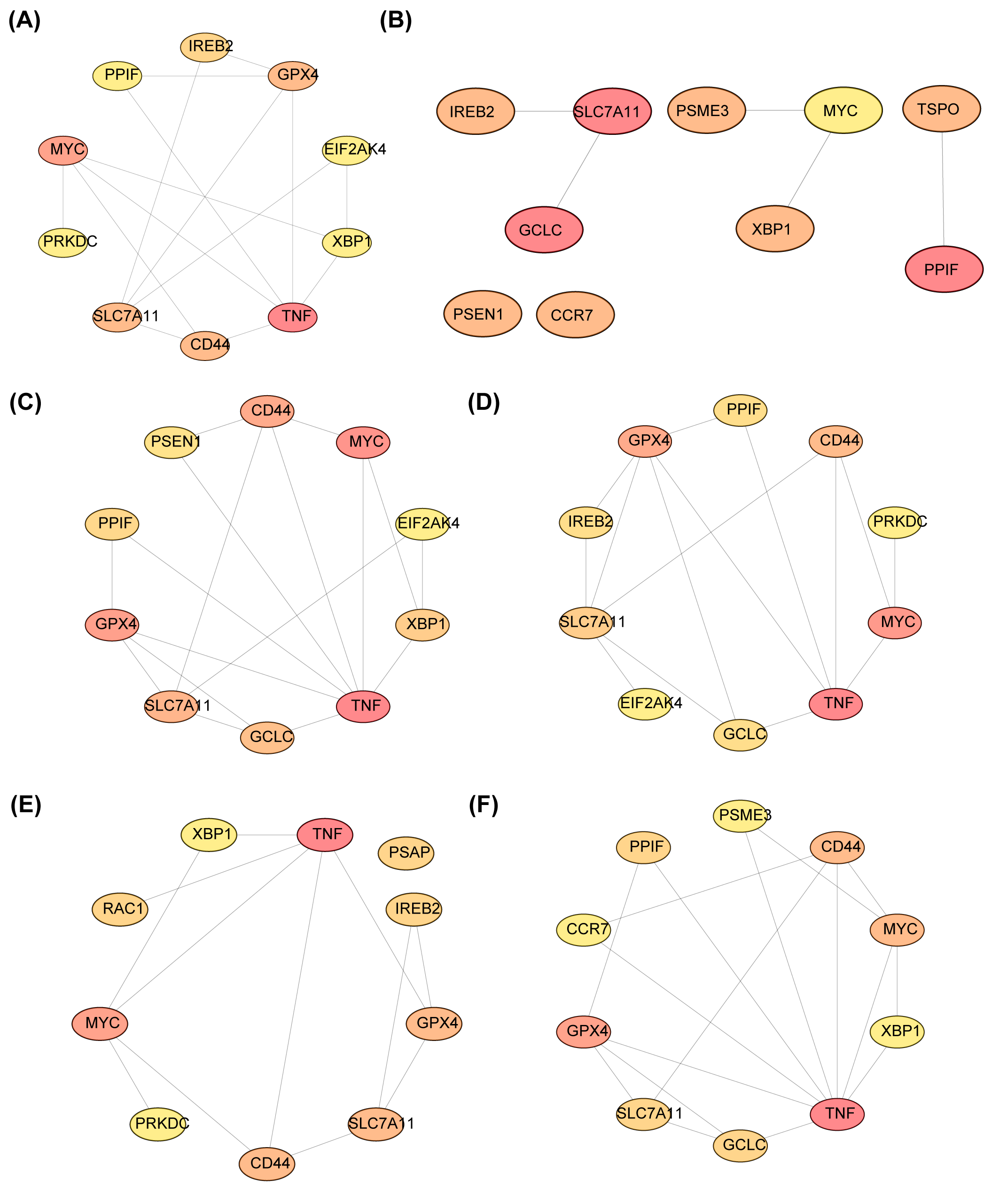

Supplement: Supplementary file 5 [file Image1.tif]
